# Sleep, physical activity and mental health among 361 French business leaders: A cross-sectional descriptive study

**DOI:** 10.1371/journal.pgph.0005880

**Published:** 2026-02-03

**Authors:** Valentin Bourlois, Pauline Baron, Charlotte Elsworth-Edelsten, Sonia Levillain, Charlotte Bonduelle, Yohan Roussel, Thierry Pezé, Rémy Hurdiel

**Affiliations:** 1 Universite Littoral Côte d’Opale, Universite Artois, Universite Lille, ULR 7369 - URePSSS - Unité de Recherche Pluri-disciplinaire Sport Santé Société, Dunkerque, France; 2 School of Human and Life Sciences, Canterbury Christchurch University, Canterbury, Kent, United Kingdom; 3 IÉSEG School of Management, Puteaux, France; PLOS: Public Library of Science, UNITED STATES OF AMERICA

## Abstract

Recent studies revealed that more than half of French business leaders are at risk of burnout. They sacrifice sleep, physical activity and often work over 60 hours weekly. Poor sleep and lack of exercise contribute to major health issues in general population. To date, no study explored the variations in health status among business leaders across different types and sizes of companies. This study aims to assess health among French business leaders, focusing on sleep quality, physical activity, anxiety, and stress levels across their different organizational contexts. We hypothesized that hierarchical positions and level of responsibility was associated with severity of health issues. 361 business leaders (158 women/203 man) agreed to complete questionnaires including: Pittsburg Sleep Quality Index, Perceived Stress Scale, Global Anxiety Disorder and International Physical Activity Questionnaire. RStudio software was utilized for descriptive statistics and analyses. Results revealed that 67.3% of them have poor sleep, 47.8% are highly stressed, and 22.5% have very low levels of physical activity. Women exhibit worse mental health and top leaders of small enterprise experience more stress, practice less physical activity and have poorer sleep The findings underscore the need for targeted health promotion strategies for leaders that take into consideration sex and organizational context.

## Introduction

Recently, the Harvard Business Review reported that more than 50% of managers feel burned out [1]. In 2022, French surveys [2] revealed that more than half of French business leaders were at risk of burnout, with 10% of them facing a very high risk. Consequently, 27% of managers have already considered suicide [3]. In 2022, burnout was recognized by the World Health Organization (WHO) under the 11th revision of the International Classification of Diseases (ICD-11) as a professional phenomenon, described as “a syndrome resulting from chronic workplace stress that has not been successfully man-aged” [4]. To manage their work-life balance, business leaders often sacrifice their sleep, physical activity, and leisure time. A recent study [5] reported that among 400 French business leaders 39% of them work more than 60 hours per week, and 49% report sleeping on average 6.5 hours per night. This is at least one hour less than recommendations for adults [6]. Research by Zawieja et al., [7] showed that commitment to a company is a vector of self-fulfilment and performance, but when it becomes excessive, it can also lead to burnout and poor global health. In 2012, the National Institute of Sleep and Vigilance [8] showed that within the 25% of French people sleeping less than six hours per night (weekdays), business leaders and independent workers were the most represented. In addition, it was found that 30% of leaders do not implement any self-care practices, 34% suffer from sleep disorders and 32% experience physical illness due to low levels of exercise [9]. Sleep and physical activity are two crucial determinants of health stability in the general population [10], suggesting that business leaders should pay particular attention to them.

One way to categorize French companies is to classify them by size based on employee count (Law on the Modernization of the Economy (LME) 2009 [11]): Very Small Enterprises (VSEs) with 0–10 employees, Small and Mediumsized Enterprises (SMEs) with 11–250 employees, Intermediate-Sized Enterprises (ISEs) with 251–5,000 employees, and Large Enterprises (LEs), any company not corresponding to the previous categories. Large enterprises, often listed on the CAC40 stock exchange, typically have hierarchical structures and multiple branches, with leadership often shared by groups of shareholders or executives. SMEs and VSEs, ranging from self-employed individuals to firms with up to 250 employees, place much of the pressure on the business leader, who is often the final decision maker. ISEs fall somewhere between these two models. The health impacts on managers of large enterprises (LE) and small to medium sized enterprises (SMEs) may differ due to variations in work environments and responsibilities. Leadership positions involve high responsibilities and intense demands, particularly differentiated by company size. In LEs and ISEs, leadership is shared through hierarchical structures and dedicated units [12], diluting responsibilities [13]. Conversely, leaders of VSEs and SMEs accumulate multiple roles (heavy workload, recruiter, salesperson, crisis decision-maker) without collegial support, generating entrepreneurial solitude, existential stress [14], and pathogenic stress linked to economic uncertainty [15–17]. This stress, higher than among salaried employees [18], exposes them to cardiovascular risks, burnout, and forgoing care [19]. Healthy and shared leadership is essential to mitigate these burdens [20,21]. These structural differences directly influence leaders’ sleep patterns and justify exploring their specific impact on sleep, physical activity, and mental health.

Leadership health directly impacts organizational performance. Sleep deprivation and chronic sleep restriction result in daytime sleepiness, attention deficit and poor neurobehavioral performance [22] critical for leaders’ decision-making under pressure. Over time, poor sleep causes health issues such as cardiovascular diseases, metabolic syndrome, mental disorders, and early risk of cancer [23–27]. Precisely the outcomes observed in VSE/SME leaders facing existential stress. Physical activity and exercise are widely recognized for their numerous benefits to both physical and mental health [28]. Current WHO guidelines recommend at least 150 minutes of moderate intensity exercise per week to maintain good health, reduce the risk of premature mortality, and improve quality of life. Physical activity plays a key role in the prevention and management of various chronic diseases, such as cardiovascular diseases, type 2 diabetes, hypertension, and certain cancers [25,29–31]. In terms of mental health, physical activity is associated with reduced symptoms of anxiety and depression [32]. It also promotes neurogenesis and enhances cognitive function, which may lower the risk of cognitive decline related to aging and diseases such as Alzheimer’s [33]. Moreover, being active contributes to better sleep quality and stress management, both of which are crucial for overall well-being [24]. Sleep quality influences engagement in daily physical activity, while exercise improves sleep [34]. Numerous studies [35] have demonstrated the positive effects of physical activity and research suggests that regular physical activity can help prevent work related stress and burnout. essential countermeasures for the leadership stressors identified above.

SME leaders might face broader responsibilities, leading to higher stress and isolation. Their lack of structural support such as wellness programs, and the concentration of managerial responsibility on their shoulders, could increase their risk of chronic stress and burnout. LE managers, though pressured by large teams and shareholder demands, may have access to better resources [36]. Therefore, in this study, we aimed to determine the extent to which these workforce scales affect the health of managers and executives, particularly about stress, sleep, and physical activity. We hypothesized that leaders’ health outcomes are impacted by their hierarchical position, company size, and gender

## Materials and methods

### Ethics statement

This study was approved by the Ethics Committee Research (CERSTAPS) under the number IRB00012476-2023-11-01-216. All participants received an information letter explaining the study purpose, procedures, and voluntary nature of participation prior to completing the questionnaire. The study was conducted in accordance with the ethical standards of the Declaration of Helsinki. A declaration No. 2227560 v 0 to the CNIL (French National Commission on Informatics and Liberty) was made.

### Study design

This observational and cross-sectional study was conducted in France, with the help of occupational health associations and company management clubs of the Haut de France region. The data were collected in 2023 and 2024, the recruitment was beginning precisely on 11/01/2023 and ending on 15/12/2024. A power analysis was conducted to determine the required sample size for a two-group independent Student’s t-test. The calculation, performed with a significance level α of 0.05 and a power (1-β) of 0.80, indicated that a total of 170 participants would be needed to detect a medium effect size difference between the two groups. The power analysis was performed using R Studio software version 4.2.76. All participants completed the questionnaires either with the investigator or online, depending on their availability (taking between 15–20 minutes).

### Participants

A sample of 361 business managers and leaders responded to a series of questionnaires related to physical activity, sleep, stress, and anxiety as described below. Gender, age, and company size were also collected.

Inclusion criteria were being between 18 and 65 years old and being a manager or a business leader according to the following definition:

Leaders: any person who runs, manages, and represents a commercial company [37]. This also includes members of management bodies (chairman of the board, board of directors, level managers, general directors, etc.). In this study, most of the time leaders are the final decision makers and are legally responsible for the actions undertaken in the name of their company. They are directly responsible for their business’ sustainability.

Managers: any person managing a team. In our study, managers can either belong to the board of directors or not, manage large or small teams. Their main distinction from the leader’s population is that there is a power dilution and a shared responsibility with other executives. They are not directly supporting their company sustainability. In this study the managers were all in ISE and LE, and leaders were in VSE and SME, so we created 2 groups, VSE & SME, and LE & ISE.

Exclusion criteria were anyone who has travelled in the month preceding the event and subsequently experienced a time difference of more than 2 hours, being night workers or shift workers and individuals undergoing medical treatment, the Leaders or managers who were no longer holding their positions, Freelancers or independent contractors who also have a separate salaried position.

### Study instruments

#### Subjective sleep.

The study utilized the French version of the Pittsburgh Sleep Quality Index (PSQI) to evaluate candidates’ sleep quality [38]. Composed of 19 questions, the PSQI is designed to differentiate good and poor sleepers by assessing seven key sleep parameters over the past month: subjective sleep quality, sleep latency, sleep duration, habitual sleep efficiency, sleep disturbances, use of sleep medication, and daytime dysfunction. Each parameter is scored on a scale from 0 to 3, where a score of 3 indicates a negative extreme. The overall PSQI score is obtained by summing these seven component scores, yielding a range from 0 to 21. According to established classifications, a global PSQI score of 5 or less signifies good sleep quality, while a score exceeding 5 indicates poor sleep quality.

#### Physical activity.

The French version of the International Physical Activity Questionnaire (IPAQ) was used to assess the level of physical activity [39]. The IPAQ evaluates the duration, frequency, and intensity of exercise. Through 7 questions, the IPAQ provides the sum of days and minutes spent engaging in vigorous physical activity (PA), moderate PA, and walking, as well as the number of minutes spent sitting on a weekday. The sum of all these elements represented each PA modality in MET-minutes/week: MET level × minutes of activity/day × days/week. This was done three times, giving a distinct total of MET-minutes/week for each modality (walking = 3.3 MET, moderate = 4.0 MET, vigorous = 8.0 MET). Thus, the IPAQ generates “low”, “moderate”, and “high” PA scores, following these recommendations: i. Low - No activity is reported OR an activity is reported but does not reach levels Moderate or High; ii. Moderate (Meets one of the following 3 criteria) - 3 or more days of vigorous activity for at least 20 minutes per day OR 5 or more days of moderate intensity activity and/or walking for at least 30 minutes per day OR 5 or more days of any combination of walking, moderate intensity or vigorous intensity activities achieving a minimum of 600 MET-minutes/week; iii. High (Meets one of the following 2 criteria) – Vigorous intensity activity on at least 3 days and accumulating at least 1500 MET-minutes/week OR 7 or more days of any combination of walking, moderate intensity or vigorous intensity activities achieving a minimum of 3000 MET-minutes/week.

#### Anxiety.

The French version of the Generalized Anxiety Disorder questionnaire (GAD-7) was used to assess the level of anxiety [40]. Anxiety is a pervasive and excessive human emotion which can generate important emotional disorder. The GAD-7 is a unidimensional instrument, consisting of 7 items assessing the presence of anxiety symptoms over the previous 14 days. It uses a Likert scale ranging from 0 to 3 (0 = not at all, 1 = several days, 2 = more than half the days, and 3 = nearly every day). The sum of the items yields a total anxiety score, ranging from 0 to 21. Severity is determined by threshold scores; 0–4 normal symptoms, 5–9 mild symptoms, 10–14 moderate symptoms, and 15–21 severe symptoms [41]

#### Stress.

The French version of the 10 item Perceived Stress Scale was used to obtain a level of stress [42]. The PSS10 consists of 10 questions about the unpredictable and uncontrollable nature of their lives during the past month. Items were scored on a Likert scale from 0 to 4. Four of the 10 questions were positively worded and therefore were reverse scored to obtain the correct overall perceived stress score. Each participant’s score may range from 0 to 40. Higher scores indicate higher perceived stress. PSS scores between 0 and 13 indicate low stress; 14–26 represent moderate stress; and 27 or above represents severe stress [43].

### Data analysis

A descriptive analysis of all variables was performed by calculating frequencies for qualitative variables and mean values, and standard deviations for quantitative variables.

Quantitative variables were described by their mean and standard deviation (SD) and were compared using a student test or a Wilcoxon test. To determine which test to use, the normality of the data and the homogeneity of their variance were verified using a Shapiro-Wilk test and a Levene test, respectively qualitative variables were described as percentages and compared using the Fisher test.

Multivariate logistic models were fitted to estimate the risk factors associated with the social position of the company with dependant variables. In this context, risk factors are variables that increase the probability of a specific outcome, here the social position of the company. The age and sex were added to the model in correction. Odds ratios (OR) were estimated with their 95% Confidence Interval (CI) to quantify the association between each potential risk factor and the dependent variable. An OR greater than 1 indicates that the factor increases the risk, while an OR less than 1 suggests a protective effect. A step-by-step variable selection procedure was carried out based on the AIC (Akaike Information Criterium) to identify the most relevant risk factors by balancing model complexity and goodness of fit. This approach allows for the identification and quantification of the relative importance of different risk factors associated with the company’s social position, while accounting for the potentially confounding effects of age and Sex. Analyses were performed using R Studio software version 4.2.76.

## Results

The sample comprised 361 business leaders (203 men, 158 women), with a mean age of 45.09 ± 10.08 years (44.3 ± 10 for women and 45.7 ± 10 for men; no significant difference). The descriptive characteristics revealed a significant difference in sex distribution between company sizes (Table 1).

**Table 1:** Sex proportions across company sizes.

	All (N = 361)	Women (N = 158)	Men(N = 203)	P -value
**Size Company**				<0.001***
*LE & ISE*	37.4	24.0	47.8	
*SME & VSE*	62.6	76.0	52.2	

Characteristics sample: percent proportion and mean ± SD, *** = p < 0.001, Ns = No significance. Sex proportions by company size (LE & ISE: Large/Intermediate-Sized Enterprises; SME & VSE: Small/Medium/Very Small Enterprises).

The descriptive analysis shows a significant difference between women and men on the PSQI, IPAQ, PSS10 and GAD7 variables (Table 2).

**Table 2:** Table of dependant variables and sex proportions.

	All (N = 361)	Women (N = 158)	Men(N = 203)	P -value
**PSQI score**	7.3 ± 3.5	8.2 ± 3.8	6.6 ± 3.1	<0.001**
**Sleep quality (%)**				
*Good (≤ 5)*	32.7	29.1	35.5	
*Poor (> 5)*	67.3	70.9	64.5	
**PA level (%, IPAQ)**				<0.05 *
*Low*	22.5	25.3	30.5	
*Moderate*	48.9	45.0	52.2	
*High*	28.6	29.7	17.2	
**PSS10 score**	26.4 ± 6.5	28.4 ± 6.0	25.0 ± 6.4	<0.01 **
**Stress level (%)**				
*No stress*	24.2	12.6	32.5	
*Moderate stress*	28	26.6	29.1	
*High stress*	47.8	60.8	38.4	
**GAD 7 score**	6.8 ± 5.3	8.3 ± 5.5	5.8 ± 5.0	<0.01 **
**Anxiety level (%)**				
*No Anxiéty*	37.1	27.2	44.3	
*Slight Anxiéty*	34.3	35.4	33.5	
*Moderate Anxiéty*	17.6	20.9	15.3	
*High Anxiéty*	11	16.5	6.9	

Dependant variable: percent proportion and mean ± SD.

* p < 0,05;** p < 0,01; *** p < 0,001; The sex difference in the scores of dependent variables and the distribution of scores specific to each variable.

**Fig 1:**
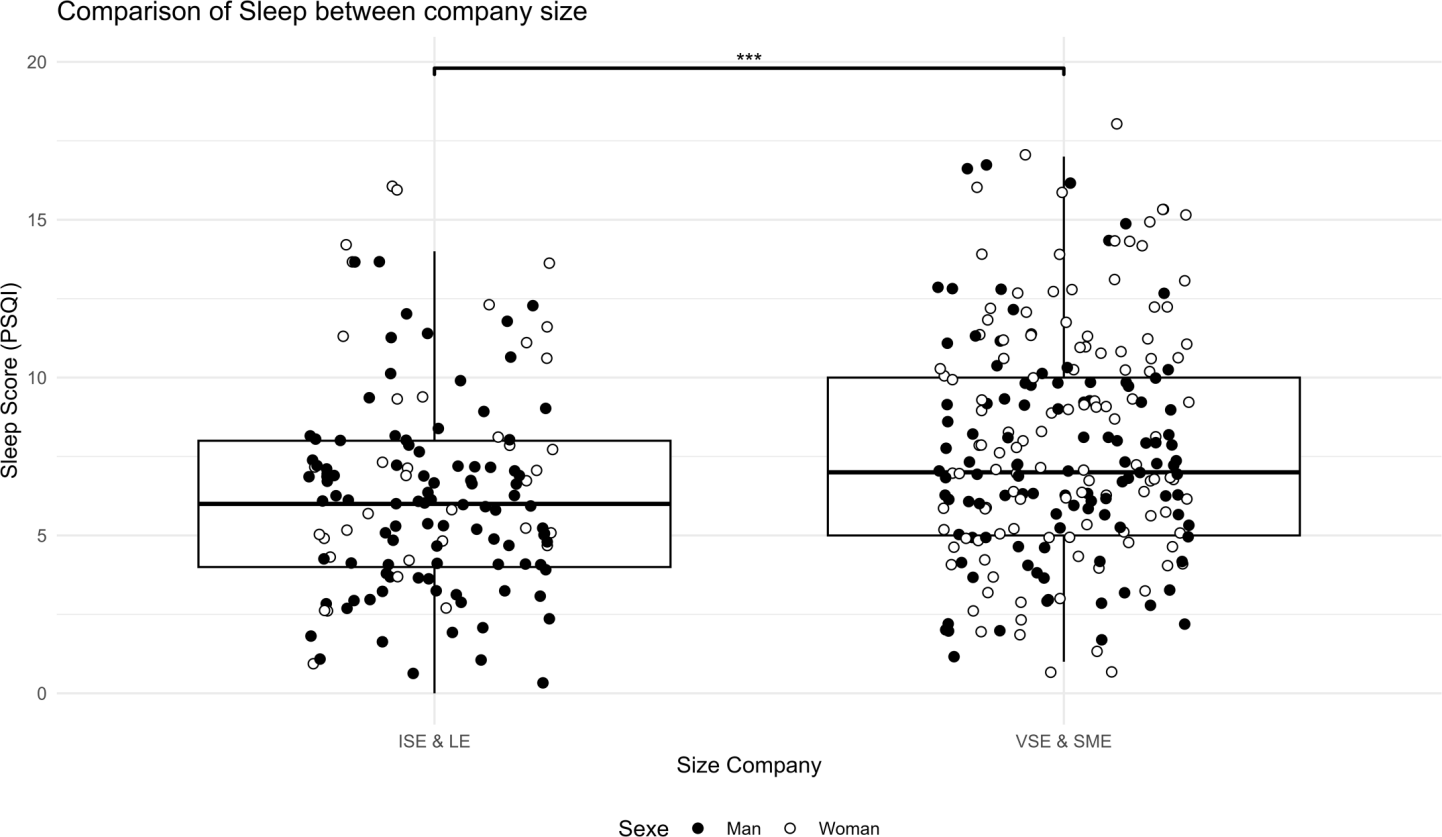
Comparison of sleep between company size. Sleep score PSQI survey comparison between size company (LE & ISE: Large Enterprise & Intermediate-Sized Enterprises; SME & VSE: Small and Medium Enterprise & Very Small Enterprise) and distribution of sex in the two sample, ISE & LE vs. VSE & SME: *** p < 0.001. PSQI score of 5 or less signifies good sleep quality.

**Fig 2:**
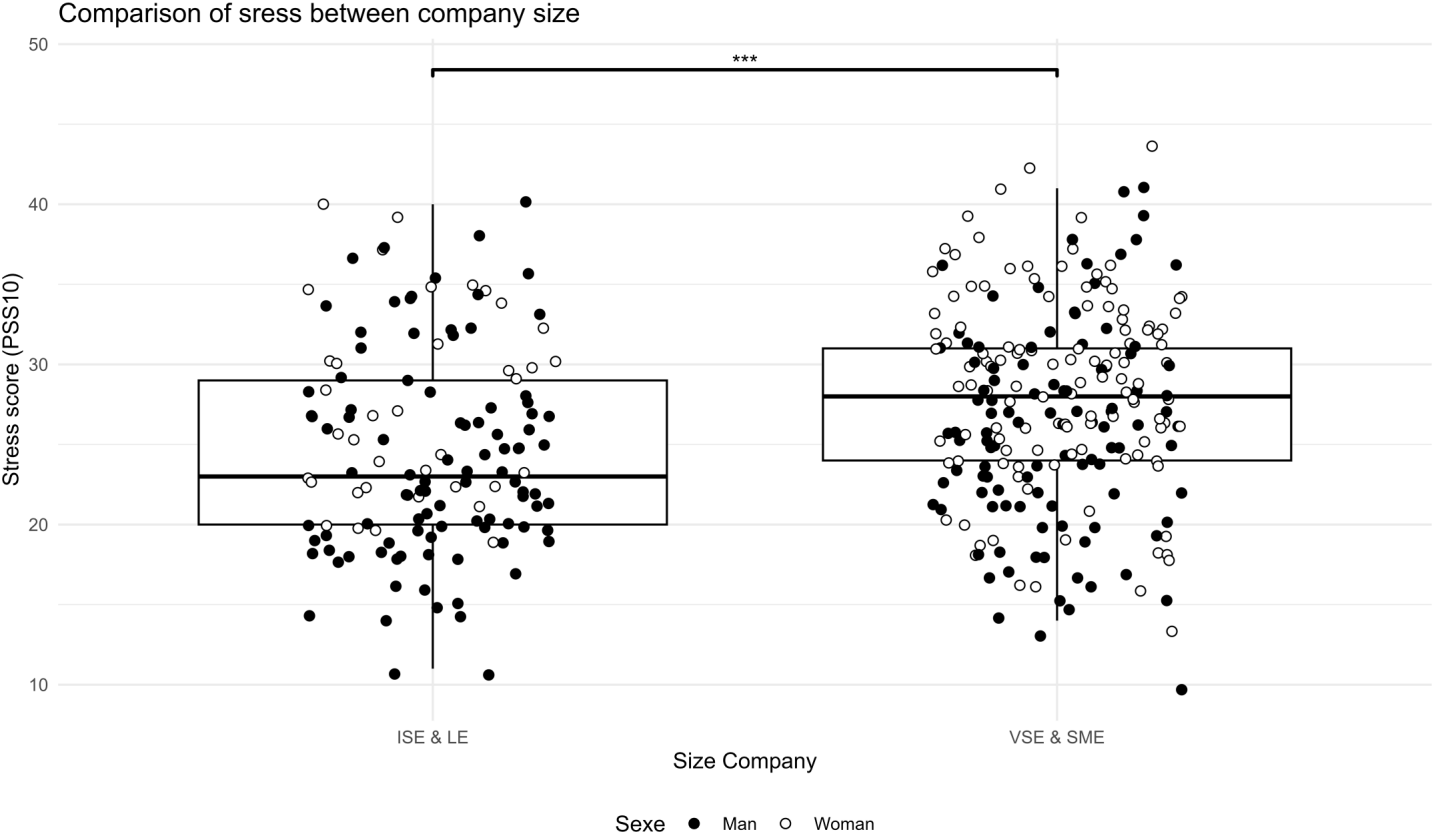
Comparison of stress between company size. Stress score PSS10 survey comparison between size company (LE & ISE: Large Enterprise & Intermediate-Sized Enterprises; SME & VSE: Small and Medium Enterprise & Very Small Enterprise) and distribution of sex in the two sample, ISE & LE vs. VSE & SME: *** p < 0.001. PSS scores between 0 and 13 indicate low stress; 14-26 represent moderate stress; and 27 or above represents severe stress.

**Fig 3:**
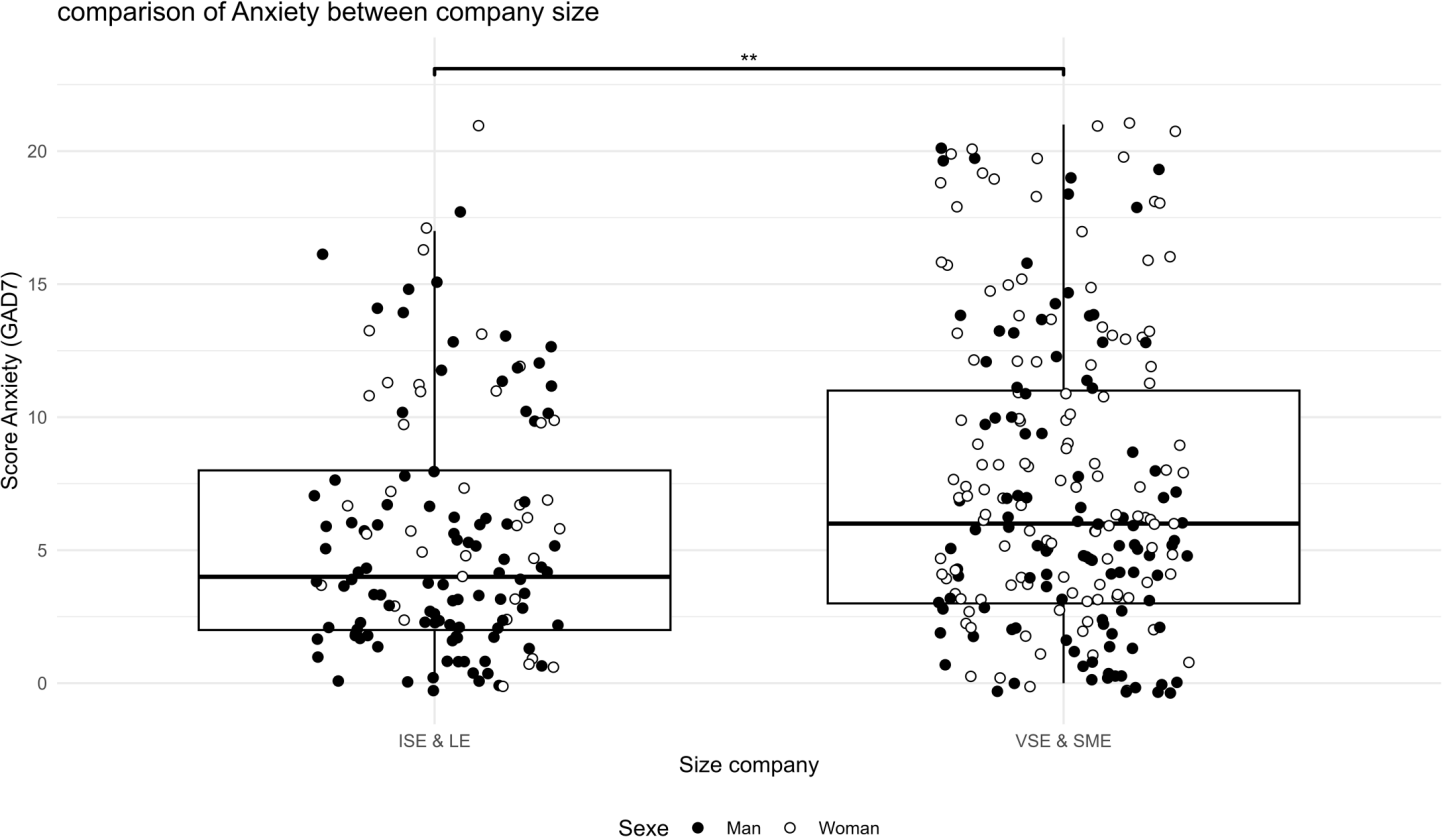
Comparison of anxiety between company size. Anxiety score GAD7 survey comparison between size company (LE & ISE: Large Enterprise & Intermediate-Sized Enterprises; SME & VSE: Small and Medium Enterprise & Very Small Enterprise) and distribution of sex in the two sample, ISE & LE vs. VSE & SME: ** p < 0.01. GAD7 scores between 0 to 4 indicate normal symptoms, 5 to 9 mild symptoms, 10 to 14 moderate symptoms, and 15 to 21 severe symptoms.

**Fig 4:**
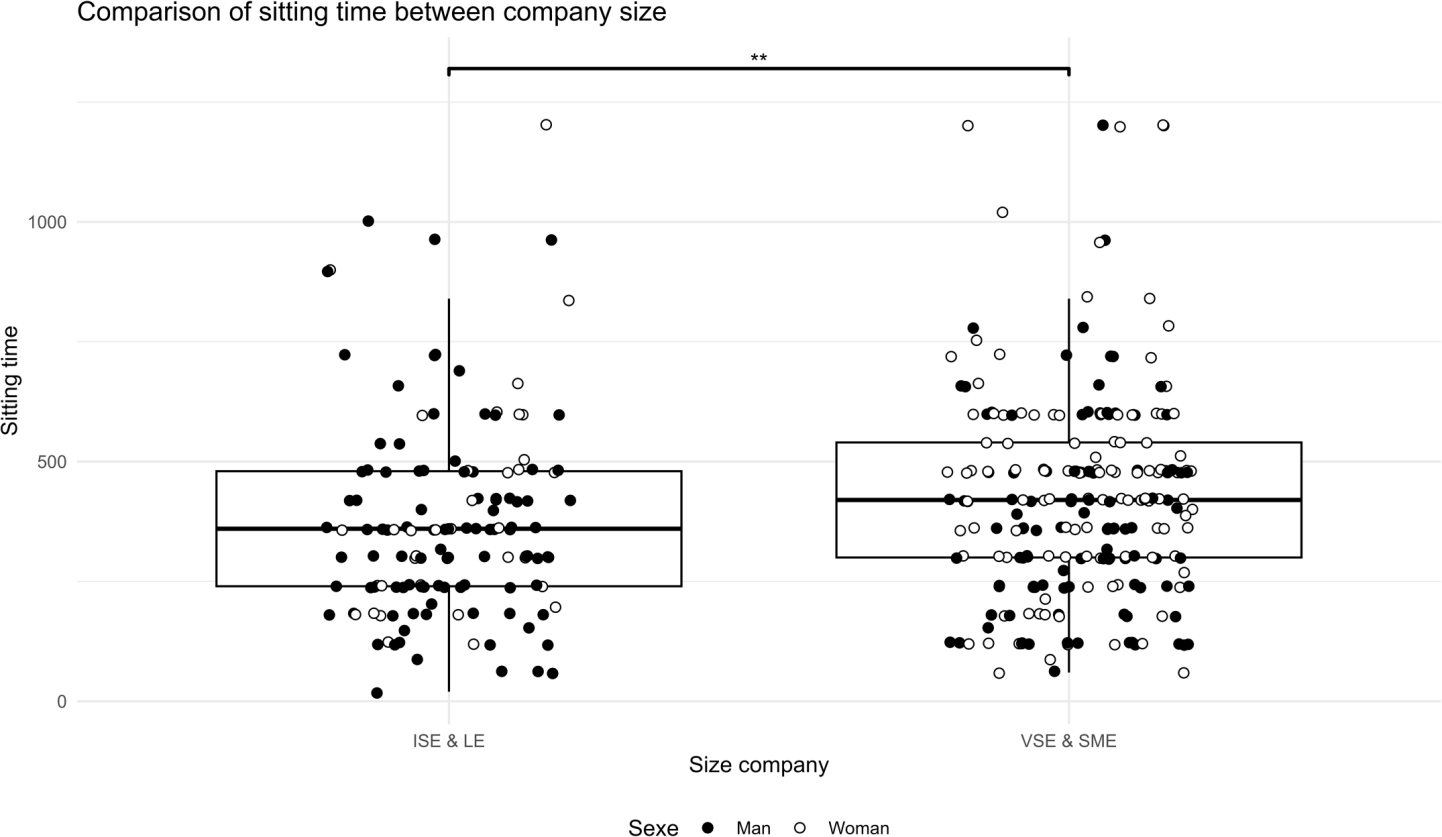
Comparison of sitting time between company size. Sitting time in IPAQ survey comparison between size company (LE & ISE: Large Enterprise & Intermediate-Sized Enterprises; SME & VSE: Small and Medium Enterprise & Very Small Enterprise) and distribution of sex in the two sample, ISE & LE vs. VSE & SME: * p < 0.05. The sitting time represented a sedentarily time.

The analyses showed a difference between SME/VSE leaders and ISE/LE managers. SME & VSE leaders were more likely to have poor sleep quality (Fig 1, t(307.8) = -3.8, p = 0.0001), higher perceived stress (Fig 2, t(279.06) = -3.6, p = 0.0003), higher anxiety (Fig 3, W = 12188, p = 0.001, r = 0.13), higher sitting time (Fig 4, W = 12861, p = 0.01, r = 0.13), and less moderate or vigorous physical activity (p = 0.001).

Sleep and stress were positively associated with the type of company, indicating a higher risk of poor sleep quality (OR = 1.82, 95% CI [1.82, 1.14], p < 0.05) and a risk of higher stress (OR = 2.01, 95% CI [1.15, 3.51], p < 0.05) among SME & VSE leaders compared to managers of LE & ISE.

Physical activity was negatively associated with the type of company, indicating decrease in moderate (OR = 0.51, 95% CI [0.27, 0.97], p < 0.05) and high (OR = 0.47, 95% CI [0.23, 0.95], p < 0.05) physical activity, among SME & VSE leaders compared to managers of LE & ISE.

Sleep was positively associated with age, indicating a higher risk of poor sleep quality with an increase in age (OR = 1.02, 95% CI [1.00, 1.05], p < 0.05). In addition, higher physical activity was negatively associated with age, indicating decrease of higher physical activity with the age increase (OR = 0.97, 95% CI [0.93, 0.99], p < 0.01).

Moderate and high stress was negatively associated with sex, which mean higher risk moderate and higher stress for women (respectively, OR = 0.45, 95% CI [0.23, 0.86], p < 0.05; OR = 0.28, 95% CI [0.15, 0.52], p < 0.001).

No other significant items.

## Discussion

This study reveals that more than half of leaders declared poor sleep, high stress, and poor physical activity habits. This is high compared to the general population [8,44], INSV estimated in 2023, 37% of French people had poor sleep [45]. With this survey, we gained insights into managers and leaders health determinants that should be useful to determine health promotion strategies among leaders.

This study highlighted that women reported poorer mental health despite a slight increase in physical activity habits compared to men. Indeed, it seems that women in leadership positions are particularly exposed to stress and sleep restriction. These results could be explained by diverse factors: professional cultures, personal beliefs, experiences and desires, psychological mindset, etc. Female leaders frequently experience a double burden, combining high responsibilities at work with domestic or family expectations, which can contribute to increased stress and poorer sleep [46]. Although norms are evolving, numerous studies show that women continue to take on a significant share of domestic tasks, even when they are in positions of responsibility. Women can also feel additional pressure to prove their competence in male-dominated environments or can experience impostor syndrome [47]. This “pressure for perfection” can lead to increased tension and work overload in order to be perceived as equally competent as men [48,49]. Some studies examining insomnia issues experienced by women demonstrated that physical activity helped improving physical capabilities and reducing sleep disorders [34]. These could suggest regarding our sample that even if women seem sensitive to the importance of physical activity for their global balance, they will need to invest even more than men in self-care routines to compensate the effects of the personal and professional pressure they experience.

Independently of the sex status, the hierarchical position, and the level of responsibility within the company also appears to influence the severity of health status. Within this sample, the influence of the company size and thus the leader position in the company was observed. Leaders of VSE and SME are more often exposed to stress and sleep deprivation than managers of ISE and LE. While age can influence sleep quality through altered architecture and weakened circadian rhythms [50], our cohort’s mean age suggests sleep deprivation culture and poor lifestyle habits as primary drivers [51]. This might be explained by different structural factors specific to small structures. VSEs and SMEs may be exposed to a high economic and cash-flow risk that can directly impact their survival. VSE and SME owners also often invest their personal resources in their business and are legally responsible for the company’s actions. Undertaking this economic pressure, financial risk, and moral and legal responsibility can affect the sleep and well-being of these leaders, especially in times of economic uncertainty [52,53]. Leaders also usually handle most of the operating aspects of the business (development, operations, finance, marketing, human resources, etc.) with little support and time for reflection. The business sustainability directly depends on their decisions, which can increase mental load. However, companies can structure their operating and decision-making model and consolidate their financial stability as they grow and their size increase.

This is what happens in ISEs and LEs managers are usually in charge of a specific perimeter and responsibilities are often shared and delegated between departments and teams. Although this population faces significant pressure, their organizations benefit from a dilution of power and responsibility, which contributes to sustainability. Managers also have the possibility to focus on their personal and professional path while fulfilling their managerial activities and responsibilities. ISEs and LEs generally have more resources, can provide more support to their managers, sometimes offer mentoring programs or healthcare facilities to their employees. However high workload, time and resources constriction, social climate, or organizational complexity can affect managers physical and mental health and work/life balance, even if the company survival is not directly in their hands [36,54].

This study also reveals that SME and VSE leaders have lower levels of physical activity than managers of ISE and LE. This could be a direct consequence of significant working hours for business leaders, about 60 hours per week according to recent surveys, which leaves very little time for physical activity [55]. Nevertheless, sedentary lifestyle is recognized to be a health risk factor. While this is not the case for our specific population of leaders, some models for reducing sedentary behaviors are being implemented as part of workplace health programs aimed at employees [56] and could be considered viable solutions for leaders.

This study has several limitations. First, the cross-sectional design precludes causal inferences; longitudinal studies are needed. Second, our survey was conducted in a single French region (Hauts-de-France), so results may not be representative of the national population or other regions/countries. Region-specific factors, such as the local economic climate, may affect responses and limit generalizability. Third, although overall mean age was 45.09 ± 10.08y, the relatively wide age range may influence physical activity, life stages, work-life balance, sleep quality, and stress levels. Age-adjusted logistic models confirm company size’s independent effect, but residual confounding from unmeasured life stage factors cannot be excluded. Finally, our inclusion criteria (leaders/managers only) may mask hierarchical influences within companies. We grouped VSE/SME leaders with ISE/LE managers based on sample composition differences.

In addition, responses may be influenced by differences in how the questions are understood, particularly concerning the IPAQ. Misinterpreted questions lead to bias and limit the reliability of the data collected. Questionnaire surveys rely on subjective, self-reported answers, which may be inaccurate, influenced by errors in memory. These limitations must be considered when interpreting the results and making recommendations.

## Conclusion

Our findings reveal the critical need for immediate action to support leaders and managers health. The intrinsic distinctive characteristics of a leader’s lifestyle might require the creation of specific programs that are adapted to their constraints to enable them to engage long term in healthier selfcare practices. Another proposal to encourage leaders to be physically active could be proposed and promoted by entrepreneurial networks. On top of raising awareness among leaders, they could organize active sessions integrated into meetings, creating new opportunities for networking while staying active. Regular sessions and collective challenges may reinforce commitment and could make exercise an accessible and enjoyable priority. The study highlights the impact of hierarchical position, company size, and gender on health outcomes. Additionally, the study acknowledges structural factors that contribute to increased stress in small business leaders, such as financial risk and workload distribution.
